# The Role of Statins in Prevention of Preeclampsia: A Promise for the Future?

**DOI:** 10.3389/fphar.2017.00247

**Published:** 2017-05-05

**Authors:** Vasiliki Katsi, Georgios Georgountzos, Manolis S. Kallistratos, Ioannis Zerdes, Thomas Makris, Athanasios J. Manolis, Petros Nihoyannopoulos, Dimitris Tousoulis

**Affiliations:** ^1^Department of Cardiology, Hippokration HospitalAthens, Greece; ^2^School of Medicine, University of PatrasPatras, Greece; ^3^Department of Cardiology, Asklepeion General HospitalAthens, Greece; ^4^Faculty of Medicine, School of Health Sciences, University of IoanninaIoannina, Greece; ^5^Department of Cardiology, Elena Venizelou HospitalAthens, Greece; ^6^First University Department of Cardiology, Hippokration Hospital, University of AthensAthens, Greece

**Keywords:** preeclampsia, hypertension, statins, pravastatin, prevention

## Abstract

Preeclampsia has been linked to high morbidity and mortality during pregnancy. However, no efficient pharmacological options for the prevention of this condition are currently available. Preeclampsia is thought to share several pathophysiologic mechanisms with cardiovascular disease, which has led to investigations for the potential role of statins (HMG CoA reductase inhibitors) in its prevention and early management. Pravastatin seems to have a safer pharmacokinetic profile compared to other statins, however, the existing preclinical evidence for its effectiveness in preeclampsia treatment has been mostly restricted to animal models. This review aims to summarize the current data and delineate the potential future role of statins in the prevention and management of preeclampsia.

## Introduction

Preeclampsia is a pregnancy-related disorder with multi-organ involvement, mainly including the emergence of high blood pressure (BP) in the second trimester of pregnancy. Affecting about 1 in 12 pregnant women in the USA, preeclampsia is the most common hypertensive condition complicating pregnancy, as well as one of the major causes of maternal and fetal complications and death (Frishman et al., 2006; American College of Obstetricians and Gynecologists and Task Force on Hypertension in Pregnancy, 2013; Lo et al., 2013; Say et al., 2014). Although many therapeutic interventions have been proposed, mortality and morbidity rates still remain considerable. The aim of this review is to assess the possible role of statins in the therapeutic management of preeclampsia through exploration of the current preclinical and clinical data.

## Clinical Presentation

Preeclampsia is characterized by the development of new onset hypertension (HTN) and the establishment of proteinuria. Other signs and symptoms that accompany the disease include: headache, visual disturbances, epigastric or abdominal pain, weakness, altered mental status, HELLP syndrome (Aloizos et al., 2013) dyspnea and edema (American College of Obstetricians and Gynecologists and Task Force on Hypertension in Pregnancy, 2013). Many factors have been found to increase the risk of preeclampsia and their association with the disease has been adequately elucidated. Previous preeclamptic pregnancy, family history of preeclampsia, late age of maternity (>40 years), multiple gestation, obesity, diabetes mellitus and history of thrombophilia have been identified as predisposing risk factors (American College of Obstetricians and Gynecologists and Task Force on Hypertension in Pregnancy, 2013). In particular, the presence of HTN and chronic renal impairment before gestation has been strongly correlated to the development of preeclampsia later during pregnancy (Foo et al., 2015).

Preeclampsia can result in a great number of severe and, in some cases, fatal short- and long-term consequences affecting both the mother and the fetus. Maternal complications include cardiometabolic disorders (diabetes, ischemic heart disease, metabolic syndrome), cerebrovascular disease (stroke, intracranial bleeding), neurologic abnormalities (eclamptic seizures) and renal impairment (Ramsay et al., 2003; Wilson et al., 2003; Haukkamaa et al., 2004; Funai et al., 2005). Fetal outcomes include intrauterine growth restriction (IUGR), prematurity and higher risk of developing HTN, obesity, metabolic syndrome, dyslipidemias, and cardiovascular disease (Lo et al., 2013; Nice guidelines, 2016).

Delivery of the fetus is the only efficient therapy (Everett et al., 2012; Gangooly et al., 2014; Nice guidelines, 2016). If the gestational age is less than 34 weeks and the BP can be sufficiently controlled with the absence of other symptoms, pregnancy can be prolonged in order to avoid prematurity complications for the fetus. The main therapeutic goal in preeclampsia is the management of HTN, aiming for SBP of 140–150 mmHg and DBP of 80–100 mmHg (Nice guidelines, 2016). Oral antihypertensive therapy including a-methyldopa, calcium channel blockers, b-blockers and labetalol, coupled with anti-platelet agents and magnesium sulfate are considered as a therapy in hypertensive disorders in order to limit maternal and fetal complications (Sandrim et al., 2008; Nice guidelines, 2016).

## Pathophysiologic Mechanisms

Even though the precise etiology of preeclampsia remains obscure, the main pathophysiological mechanisms include maternal endothelial dysfunction (Maynard et al., 2003; Levine et al., 2004) and placental vascular impairment (Redman and Sargent, 2005; Burton et al., 2009; Brosens et al., 2011). Endothelial dysfunction has been correlated with abnormalities in pregnant lipid profile, with high levels of triglycerides. LDL oxidation, oxidative stress and increase of reactive oxygen species (ROS) play a key role in placental vascular dysfunction (Costantine and Cleary, 2013). In early onset disease, an adverse remodeling of spiral arteries, in which endothelial cells are partially replaced by cytotrophoblast cells, may cause malperfusion of the placenta (Burton et al., 2009; Brosens et al., 2011). As a result, ischemic injury and consequent inflammatory response will be generated.

The integrity of endothelial cells is crucial for the maintenance of angiogenic balance. Any endothelial damage such as deficient spiral artery remodeling mentioned above, can lead to placental oxidative stress and imbalance in the production of vasoconstrictive and vasodilative factors. Anti-angiogenic factors, such as soluble Fms-like tyrosine kinase (sFlt-1) and soluble endoglin (sEng), have been shown to increase in preeclamptic gestations (Hertig et al., 2004; Levine et al., 2004; Wang et al., 2009). Specifically, sFlt-1 blocks the binding of vascular endothelial growth factor (VEGF) and placental growth factor (PIGF) to their receptors, resulting in reduction of nitric oxide (NO) synthesis through inactivation of endothelial NO synthase (eNOs) (Shen et al., 1999; Noris et al., 2005). The low levels of NO invert the beneficial impact of vasodilatation in preventing placental ischemia (Sladek et al., 1997; Venkatesha et al., 2006; Ramma and Ahmed, 2014). Inhibition of NO production can be caused through an asymmetric dimethylarginine (ADMA)-mediated eNOS inactivation (Ehsanipoor et al., 2013). Another pathophysiologic mechanism in preeclampsia is the hemeoxygenase/carbon monoxide (HO-1/CO) pathway (Ozen et al., 2015; Zenclussen et al., 2015). Hemeoxygenase 1 has anti-inflammatory and vasoprotective properties and its down regulation seems to lead to sFlt-1 and sEng overexpression. The result of this angiogenic imbalance is the endothelial dysfunction and the appearance of the maternal clinical syndrome.

## The Potential Role Of Statins

**FIGURE 1:**
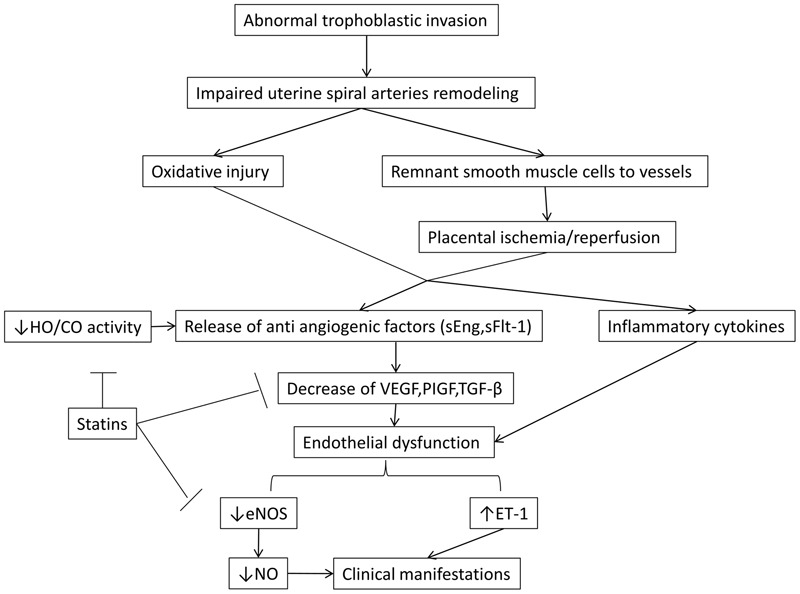
**The malfunction in the invasion of trophoblastic cells is thought to be fundamental in the cascade of preeclampsia.** Also, the role of NO and HO/CO activity in the release of antiangiogenic factors sFlt-1 and sEng is crucial in the pathogenesis of the disease. By blocking these regulatory points, statins beneficially allow the synthesis of NO by eNOS, leading to vasodilation and decrease of the secretion of antiangiogenic factors. The latter effect results in the increase of VEGF and PIGF which are key components in placental and endothelial integrity. eNOS, endothelial nitric oxidase synthase; ET -1, endothelin 1; HO-1/CO, hemeoxygenase -1/ carbon monoxide; NO, nitric oxide; PIGF, placental growth factor; sEng, soluble endoglin; sFlt-1, soluble Fms- like tyrosine kinase; TGF – β, transforming growth factor β; VEGF, vascular endothelial growth factor.

Statins inhibit HMG-CoA reductase leading to increase of LDL receptors (LDLr) and thus, to the reduction of plasma cholesterol levels. The most serious adverse effects include rhabdomyolysis and transaminase elevation but their incidence is about 1%. Statin administration has been shown to be safe in pregnancy (Kazmin et al., 2007; Ofori et al., 2007; Taguchi et al., 2008) presenting a protective role in endothelial function (Laufs et al., 1997). Beyond lowering cholesterol levels, they also have other pleiotropic actions such as antioxidant, anti-inflammatory and anti-thrombogenic effects. They have been shown to increase eNOs activity *in vitro* and *in vivo*, resulting into the overexpression of vasodilating factor NO (Laufs et al., 1997, 1998; Endres et al., 1998; Fox et al., 2011). Moreover, statins induce the activity of HO-1/CO pathway and reduce platelet aggregation (Grosser et al., 2004; Lee et al., 2004) (**Figure 1**).

The preclinical evidence supporting the use of pravastatin in the treatment of preeclampsia is currently only limited to animal models and it has focused on simulation of endothelial pathophysiologic changes in preeclamptic mice. In these studies pregnant mice were injected with adenovirus carrying sFlt-1, a factor responsible for many clinical manifestations of preeclampsia. Subsequently, a statin was administered and preeclampsia-related markers, such as sFlt-1 and sEng were monitored. Pravastatin was the most commonly used agent in the majority of preclinical animal models due to its biochemical profile, namely hydrophilic and hepatoselective properties.

Ahmed et al. (2010) focused on depicting the features of human preeclampsia at rodents, such as albuminuria and endotheliosis, by presenting their prototype CBA/J × DBA/2 mice. When treated with pravastatin CBA/J × DBA/2 demonstrated decreased levels of sFlt-1, elevated levels of VEGF and decreased hypersensitivity to Angiotensin II. Saad et al. (2014) showed that pravastatin diminished the increase of sFlt-1 and sEng in their mice model and observed a down regulation in hypoxia inducible factor 1a (HIF- 1a) and in placental TGF-β. Fox et al. (2011) concentrated on the impact of pravastatin in vascular function by measuring the levels of eNOS protein. They reached to the conclusion that pravastatin increased the levels of eNOS in the aorta and reduced the levels of sFlt-1. The animal studies of Kumasawa et al. (2011) were of paramount importance in depicting the role of pravastatin in preeclampsia. They focused on the augmentation of PIGF levels, which seemed to play an important role in improving glomerular function, reducing BP and sFlt-1 levels. Costantine et al. (2010) affirms that pravastatin administration improved endothelial function by reducing sFlt-1 and amplifying NOS expression. They also suggested that pravastatin plays an important role in prevention of IUGR in offspring’s. Except from the CBA/J × DBA/2 model, Singh et al. (2011) presented a C1q-deficient mouse model that mimics human PE. When treated with pravastatin, the pregnant C1q-deficient mice restored their placental blood flow and the angiogenic balance. Bauer et al. (2013) with their experiments in rat models with placental ischemia- induced hypertension, diverged themselves from the other investigators, not only for his mouse model but also for noticing some defects of pravastatin. They reported elevated levels of VEGF and decreased levels of sFlt-1, complying with the results of other investigators. Although they observed a restoration of antioxidant capacity, there wasn’t a restoration in angiogenic potential of serum as estimated by an endothelial tube formation assay, which is an essential disadvantage. **Table 1** summarizes the results of the animal studies reported above.

**Table 1:** Effects of pravastin in animal models.

Preclinical model	Agent	Results	Reference
CBA/J × DBA/2 mice	Pravastatin (20 ug/kg)	↓ sFlt-1 ↓ Hypersensitivity to Ang II ↑ VEGF	Ahmed et al., 2010
CD-1 mouse injected with adenovirus carrying sFlt-1	Pravastatin (5 mg/kg/d)	↓sFlt-1 Restoration of glucose response in females	McDonnold et al., 2014
CD-1 mouse injected with adenovirus carrying sFlt-1	Pravastatin (5 mg/kg/d)	Regularization of impaired vestibular function, balance and coordination linked with preeclampsia	Carver et al., 2014
CD-1 mouse injected with adenovirus carrying sFlt-1	Pravastatin (5 mg/kg/d)	↓ sFlt-1 ↓ sEng ↓ Overexpression of TGF-β in placenta ↓ HIF-1α	Saad et al., 2014
CD-1 mouse injected with adenovirus carrying sFlt-	Pravastatin (5 mg/kg/d)	↑ eNOS in the aorta	Fox et al., 2011
CD-1 mouse injected with adenovirus carrying sFlt-1	Pravastatin (5 mg/kg/d)	↓ sFlt-1 ↑ PIGF ↓ Hypertension ↓ Proteinuria	Kumasawa et al., 2011
CD-1 mouse injected with adenovirus carrying sFlt-1	Pravastatin (5 mg/kg/d)	↓ sFlt-1 ↓ Contractile response to phenylephrine ↑ Vasorelaxant response to ACh	Costantine et al., 2010
C1q deficient (C1q^-/-^) mouse	Pravastatin (5 mg/d)	↑ VEGF ↓ sFlt-1 ↓ Albumin creatinine ratio (ACR) ↓ STAT-8 ? Matrix metalloproteinase (MMP) activity Normal aortic ring response to AngII	Singh et al., 2011
Reduced utero-placental perfusion pressure (RUPP) rats	Pravastatin (1 mg/kg/d)	↓ MAP ↓ sFlt-1 ↑ VEGF ↓ sFlt-1/VEGF ratio ↓ Thiobarbituric acid reactive substances ↑ Total antioxidant capacity ↓ Endothelial tube formation No effect on HO-1 expression	Bauer et al., 2013

Studies in mice focused also on the neonatal/offspring benefit. McDonnold et al. (2014), apart from reduced sFlt-1 levels with pravastatin treatment, underlined the role of maternal treatment with pravastatin in restoration of glucose response and post-partum growth. They suggested that the effect of pravastatin in metabolic changes took place only in female offspring’s. Carver et al. (2014) supported that pravastatin administration during pregnancy in murine models ameliorated the neuromotor dysfunction by optimizing vestibular function and balance. They also showed that statins promoted the expression of genes pertained to myelination and oxidative stress, especially in male offspring’s.

Moreover, a few case reports in humans have been recently published. A case of a 30-year-old woman with known antiphospholipid syndrome, thrombosis and preeclampsia at the 23rd week of gestation has been described (Lefkou et al., 2014). The patient was treated with combined medication including pravastatin (20 mg), enoxaparin (0.4 BD) and aspirin (100 mg OD). BP and proteinuria improved and normalization of previous pathological Doppler findings of the uterine arteries were noticed. In another study (Brownfoot et al., 2015) of four patients, presented with hypertension, proteinuria, preeclampsia, and growth restricted fetuses before the 30th week of gestation, the daily administration of pravastatin (40 mg), resulted in reduced levels of sFlt-1 and stabilization of impaired glomerular function and hypertension.

A double blind, randomized placebo-controlled, multicenter trial of pravastatin to ameliorate early onset pre-eclampsia (StAmP) is currently recruiting and investigators focus on the impact of pravastatin in the reduction of anti-angiogenic markers in women with early preeclampsia (Clinical trials register.eu, 2009). Additionally, in the Pravastatin for Prevention of Preeclampsia trial, a phase I interventional trial under progress, preliminary data suggest safe pharmacokinetic profile of pravastatin with no serious adverse effects for the fetus or the mother in high-risk pregnancy (Clinical Trials.gov, 2016; Costantine et al., 2016).

## Conclusion

Preeclampsia remains a pregnancy-related health issue with severe and life-threatening complications and clinicians should be alerted for early recognition, prompt diagnosis, prevention and effective treatment. Preliminary data suggest that statins can be a promising therapeutic alternative for the prevention and treatment of preeclampsia, however, larger clinical studies are still required.

## Author Contributions

All authors listed, have made substantial, direct and intellectual contribution to the work, and approved it for publication.

## Conflict of Interest Statement

The authors declare that the research was conducted in the absence of any commercial or financial relationships that could be construed as a potential conflict of interest.
